# Systematic pan-cancer analysis reveals OGT and OGA as potential biomarkers for tumor microenvironment and therapeutic responses

**DOI:** 10.1016/j.gendis.2023.101089

**Published:** 2023-09-09

**Authors:** Chunyan Hou, Ci Wu, Wenge Zhu, Huadong Pei, Junfeng Ma

**Affiliations:** Department of Oncology, Lombardi Comprehensive Cancer Center, Georgetown University, Washington, DC 20007, USA; Department of Biochemistry and Molecular Medicine, School of Medicine and Health Science, George Washington University, Washington, DC 20052, USA; Department of Oncology, Lombardi Comprehensive Cancer Center, Georgetown University, Washington, DC 20007, USA

*Protein O*-linked β-*N*-acetylglucosamine (*O*-GlcNAc) modification (*O*-GlcNAcylation) is a unique monosaccharide modification of essential importance in physiology and pathology.^1^^,^^2^ As a highly dynamic process, O-GlcNAcylation is mediated by two paired enzymes: O-GlcNAc transferase (OGT) and O-GlcNAcase (OGA), which add or remove the modification from proteins, respectively. Emerging evidence demonstrates that the aberrant *O*-GlcNAcylation underlies the initiation, progression, and metastasis of cancer.^3^^,^^4^ Remarkably, individual studies suggest that *O*-GlcNAc cycling enzymes and *O*-GlcNAcylation hold promise as biomarkers and therapeutic targets for certain types of cancers.^1^ However, integrated and pan-cancer analysis about the key enzymes across tumors has been lacking. In this study, we systematically explored the relationship between OGT/OGA expression with pathological status and their prognostic, immunological, and therapeutic roles in various cancers. It was revealed that OGT/OGA expression levels were significantly associated with a number of tumors, immune infiltrates and immunocytes, and cancer therapeutics (including chemotherapy and immune checkpoints). Taken together, by comprehensive pan-cancer analysis of OGT and OGA, we show that OGT/OGA can serve as valuable biomarkers for multiple types of cancers, such as colon adenocarcinoma (COAD).

We first explored the differential gene expression of OGT/OGA in normal tissues and the 33 types of cancers in the TCGA datasets. OGT was found significantly changed in 16 cancer types (including COAD), while OGA showed significant differential expression in 12 tumors (Fig. 1A). Specifically, OGT and OGA were over-expressed in ten cancers and six cancers, respectively. The lower expression of both OGT and OGA was observed in five cancers (*i.e.*, BRCA, GBM, KICH, THYM, and UCEC). In line with mRNA levels, the protein levels of OGT and OGA were also found significantly changed in different types of cancer (Fig. S1). Of note, a slight discrepancy was observed between mRNA levels and protein abundances in several types of tumors. For example, distinct from OGT (Fig. 1B), OGA protein levels were not strikingly changed in primary COAD (Fig. S1). Furthermore, significant changes in the expressions of OGT and OGA were observed in different pathological stages and metastatic status of several types of cancers (Fig. S2, 3). It appeared that OGA levels are significantly correlated with the pathological status of COAD (Fig. 1C). These results suggest that the expression of OGT and OGA may be closely involved in the occurrence and progression of different tumors. We then investigated the prognostic value of OGT/OGA expression. The expression of OGT/OGA was linked to survival differences in several types of cancers (Fig. S4).

**Figure 1:**
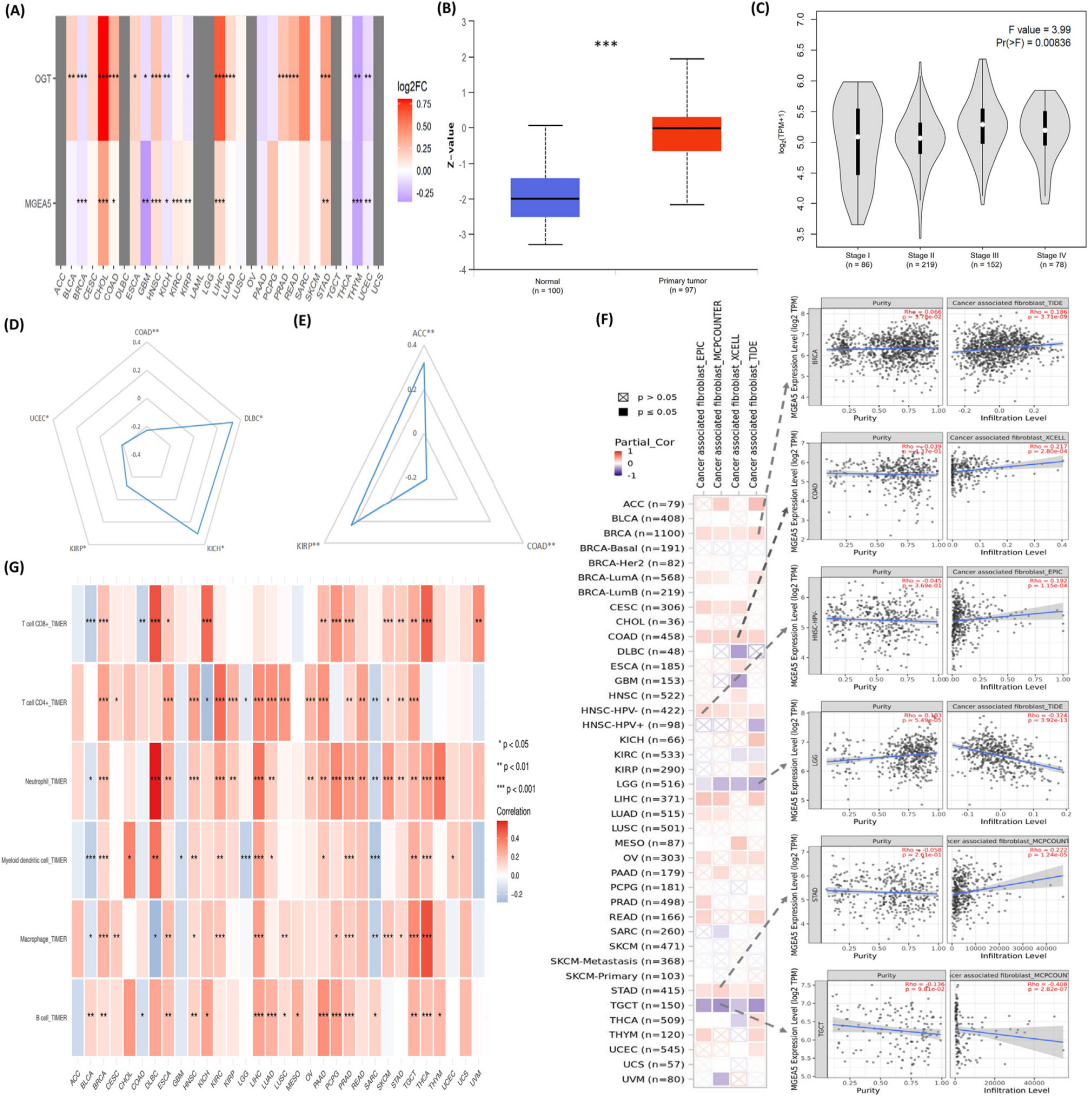
Expression of *O*-GlcNAc cycling enzymes (*i.e.*, OGT and MGEA5/OGA) and correlation with tumor microenvironment as well as immune functionality in COAD and other human cancers. **(A)** The mRNA expression levels of OGT and MGEA5/OGA in cancers and adjacent normal tissues. The color of each box represents the log_2_FC value. The statistical significance is represented by *P* values using the Wilcoxon test. ^∗^*P* < 0.05, ^∗∗^*P* < 0.01, ^∗∗∗^*P* < 0.001. Of note, grey means cancers without paired normal expression data. **(B)** Expression levels of OGT protein in COAD (*n* = 100) and normal tissues (*n* = 97). **(C)** The expression levels of MGEA5/OGA in different pathological stages of COAD. **(D)** Expression levels of OGT and tumor mutation burden (TMB) in cancers. Cancers with significant correlations are shown (^∗^*P* < 0.05, ^∗∗^*P* < 0.01, ^∗∗∗^*P* < 0.001). Positive values and negative values mean positive and negative associations, respectively. **(E)** Expression levels of OGT and microsatellite instability (MSI) in cancers. Cancers with significant correlations are shown (^∗^*P* < 0.05, ^∗∗^*P* < 0.01, ^∗∗∗^*P* < 0.001). **(F)** Expression levels of MGEA5/OGA levels with cancer-associated fibroblasts (CAFs) by using four algorithms (*i.e.*, EPIC, MCPCOUNTER, XCELL, and TIDE) across cancers (left panel). The color of each box represents the partial Spearman's correlation coefficient (Rho). The scatter plots show COAD and other cancers with significant correlations (*P* < 0.05) between expression level and CAFs, with the highest absolute coefficient illustrated (right panel). **(G)** Expression levels of OGT with the infiltration levels of various immune cells (including T cell CD8^+^, T cell CD4^+^, neutrophil, myeloid dendritic, macrophage, and B cells) in COAD and other cancers. ^∗^*P* < 0.05, ^∗∗^*P* < 0.01, ^∗∗∗^*P* < 0.001.

To evaluate the relationship between OGT/OGA level and mutations in malignancies, we explored the tumor mutation burden (TMB) and microsatellite instability (MSI) of cancers. It appears that the expression levels of OGT/OGA were remarkably linked with TMB and MSI in several tumors, albeit with either positive or negative correlations (Fig. S5). Interestingly, OGT levels appeared to be negatively related to TMB and MSI in COAD (Fig. 1D, E). As TMB and MSI are two tumor mutation mechanisms associated with immunotherapy, we reasoned that the expression levels of OGT/OGA may be highly involved in immune responses. Thus, we investigated the relationship between OGT/OGA expression and several aspects of the tumor microenvironment (including stroma score, immune score, stromal cells, and immune cells). An overall negative correlation between OGT and the stroma scores as well as the immune scores was observed in 12 out of the 33 cancers (Fig. S6A, B). Of note, none of the cancers showed a consistently significant correlation of OGT levels with cancer-associated fibroblasts (CAFs) (Fig. S7). However, the estimated infiltration value of CAFs was significantly correlated with the expression of OGA: positively in several tumors (including BRCA, COAD, and HNSC-HPV^−^) and negatively in LGG and TGCT (based on all or most algorithms; Fig. 1F). For example, the expression level of OGA in COAD was positively correlated with the infiltration level of CAFs (cor = 0.217, *P* = 2.80e−04; based on the XCELL algorithm). As important components of the tumor microenvironment, infiltrating immune cells are frequently associated with tumor behavior, drug resistance, and patient outcomes. Therefore, we studied the impact of OGT/OGA expressions on the six immune infiltrates across different cancers. Both OGT and OGA levels were significantly correlated to most immune infiltrates (*i.e.*, T cell CD8^+^, T cell CD4^+^, neutrophil, myeloid dendritic, macrophage, and B cells) except in several others (including COAD) (Fig. 1G; Fig. S8). These data suggest that both OGT and OGA are closely related to tumor microenvironment (especially immune infiltration) which may modulate tumor development and therapeutic responses.

Interventions (*e.g.*, chemotherapeutics, immunotherapy, microbiome-based therapies, and therapeutic diets) have been quickly evolving to improve cancer treatment. We explored the potential relationship of OGT/OGA expressions with cancer treatment, especially immunotherapy and chemotherapy. An overall positive correlation was observed between OGT and most of the immunoinhibitors and the immunostimulators in almost all the cancers studied (Fig. S9, 10). Similarly, OGA levels were positively associated with at least one of the checkpoints in almost all the cancers studied (except LGG) (Fig. S9, 10). For example, both OGT and OGA showed a strong positive correlation with the two immune-checkpoint inhibitors CTLA4 and PDCD1 (PD-1) in many types of cancers (including COAD), albeit with slightly different correlation coefficients and *p* values. Although immunotherapy represents a promising approach to strengthening the body's anti-tumor immune responses, only a portion of patients respond to immunotherapeutic treatment. Thus, the predictive power of OGT or OGA as a biomarker in the public immunotherapy cohorts was evaluated by the area under the receiver operating characteristic curve (AUC). OGT and OGA gave an AUC value greater than 0.5 (random) in eight and nine immune checkpoint blockade sub-cohorts, respectively (Fig. S11), suggesting the potential of applying them as predictive biomarkers. In addition, we compared the differences in drug response (IC_50_ value) for 24 anticancer drugs between groups with high- and low-expression levels of OGT or OGA. The results showed that the IC_50_ values of nine drugs were significantly varied with different OGT levels (Fig. S12A), *i.e.*, L-685458 (a γ-secretase inhibitor), PD-0332991 (an inhibitor of CDK4/6 kinases), topotecan (a topoisomerase inhibitor), sorafenib (an inhibitor of several kinases), paclitaxel (a mitotic inhibitor), panobinostat (a deacetylase inhibitor), PF2341066 (a cMet/ALK inhibitor), PLX4720 (a BRAFV600E inhibitor), and TAE684 (an ALK inhibitor). Of note, although the OGT level is not responsive to the treatment of irinotecan (a commonly used drug for stage IV colon cancer), it shows remarkable responses to the treatment of PD-0332991 (a drug in phase II clinical trial in patients with colorectal cancer). For groups with different levels of OGA, significant differences in terms of IC_50_ values were found in five drugs (Fig. S12B), *i.e.*, sorafenib, paclitaxel, topotecan, PD-0325901 (a non-ATP competitive MEK inhibitor), and L-685458. Collectively, these results suggest a strong correlation between OGT/OGA expressions and immunotherapy as well as chemotherapy drug treatment. Of note, OGT/OGA may also be involved in treatment resistance (*e.g.*, acquired by chemotherapy and radiotherapy for colorectal cancer).^5^ Thus OGT/OGA may serve as sensitive and reliable therapeutic response biomarkers to benefit selecting suitable treatment methods in clinical practice. Given the complex nature of therapies and therapeutic resistance, it is worth exploring the roles of OGT/OGA and how OGT/OGA affects the treatment of specific cancer types.

In conclusion, we conducted a comprehensive pan-cancer analysis of OGT and OGA, two enzymes in the O-GlcNAc cycling. We found that OGT/OGA expression is closely related to tumor progression and metastasis in various cancers. Moreover, OGT and OGA levels are significantly associated with immune-infiltrating levels and tumor immunity (including colorectal cancer). In addition, it appears that OGT/OGA expression strongly correlates with therapeutic responses. Of note, OGT/OGA expression discrepancies may exist between the pan-cancer analysis and individual studies using different samples (*e.g.*, cancer cell lines *vs*. patient samples, different cancer subtypes, and different stages of cancer). Although individual studies focusing on the roles of OGT/OGA in specific cancers (*e.g.*, colorectal cancer) are still lacking, further research will emphasize the importance of OGT/OGA (*e.g.*, as predictive markers) in tumor development and treatment. We hope this study provides new ideas about targeting OGT and OGA for translational applications (such as biomarkers and therapeutic targets) in the future.

## Conflict of interests

No competing financial interests were declared.

## Funding

This work was partially supported by the 10.13039/100000002National Institutes of Health (10.13039/100000002NIH)/the 10.13039/100000054National Cancer Institute (10.13039/100000054NCI) (No. P30 CA051008).

## References

[1] Ma J., Wu C., Hart G.W. (2021). Analytical and biochemical perspectives of protein O-GlcNAcylation. Chem Rev.

[2] Zachara N.E., Akimoto Y., Boyce M., Hart G.W., Hart G.W., Akimoto Y. (2022 (Chapter 18)). *Essentials of Glycobiology*.

[3] de Queiroz R.M., Carvalho Ã., Dias W.B., O-GlcNAcylation (2014). The sweet side of the cancer. Front Oncol.

[4] Hanover J.A., Chen W., Bond M.R. (2018). *O-*GlcNAc in cancer: an Oncometabolism-fueled vicious cycle. J Bioenerg Biomembr.

[5] Zhou Y., Zhang Y., Peng C. (2022). And-1 O-GlcNAcylation regulates homologous recombination repair and radioresistance in colorectal cancer. Clin Transl Med.

