# Supplementary material for: Systematic pan-cancer analysis reveals OGT and OGA as potential biomarkers for tumor microenvironment and therapeutic responses
Source: Genes Dis. 2023 Sep 9;11(4):101089. doi: 10.1016/j.gendis.2023.101089 (PMC10865246; doi:10.1016/j.gendis.2023.101089)
Supplement: Multimedia component 1 [file mmc1.pdf]

Supplementary Data

Systematic pan-cancer analysis reveals OGT and OGA as potential biomarkers for tumor microenvironment and therapeutic responses

Chunyan Hou,[a] Ci Wu,[a] Wenge Zhu,[b] Huadong Pei,[a] Junfeng Ma[a,*]

[a] Department of Oncology, Lombardi Comprehensive Cancer Center, Georgetown University, Washington DC 20007, USA.

[b] Department of Biochemistry and Molecular Medicine, School of Medicine and Health Science, George Washington University, Washington DC 20052, USA.

[*]To whom correspondence should be addressed: Tel: +1-202-6873802; e-mail: junfeng.ma@georgetown.edu

Materials and Methods

OGT/OGA expression analysis

The gene expression data used in this study were based upon The Cancer Genome Atlas (TCGA) research Network (http://www.cancer.gov/tcga) and the Genotype-Tissue Expression (GTEx) project (https://gtexportal.org/home/). We focused on 33 tumors, including adreno cortical carcinoma (ACC), bladder urothelial carcinoma (BLCA), breast invasive carcinoma (BRCA), cervical squamous cell carcinoma (CESC), cholangiocarcinoma (CHOL), colon adenocarcinoma (COAD), lymphoid neoplasm diffuse large B cell lymphoma (DLBC), esophageal carcinoma (ESCA), glioblastoma multiforme (GBM), brain lower grade glioma(LGG), head and neck squamous cell carcinoma (HNSC), kidney chromophobe (KICH), kidney renal clear cell carcinoma (KIRC), kidney renal papillary cell carcinoma (KIRP), acute myeloid leukemia, liver hepatocellular carcinoma (LIHC), lung adenocarcinoma (LUAD), lung squamous cell carcinoma (LUSC), mesothelioma (MESO), ovarian serous cystadenocarcinoma (OV), pancreatic adenocarcinoma (PAAD), pheochromocytoma and paraganglioma (PCPG), prostate adenocarcinoma (PRAD), rectum adenocarcinoma (READ), sarcoma (SARC), skin cutaneous melanoma (SKCM), stomach adenocarcinoma(STAD), testicular germ cell tumors (TGCT), thyroid carcinoma (THCA), thymoma (THYM), uterine corpus endometrial carcinoma (UCEC), uterine carcinosarcoma(UCS), and uveal melanoma (UVM). The differential expressions of OGT and MEGA5/OGA between TCGA tumor tissues and the adjacent normal tissues were retrieved by the Gene_DE module of TIMER2.0.[1] The log2 fold change and t-test p value were calculated for each of the cancer. A heatmap was plotted with the help of R package ‘ggplot2’. The statistical significance was evaluated by the Wilcoxon test. The protein expressions used in this study were from Clinical Proteomic Tumor Analysis Consortium (CPTAC), which was accessed through the UALCAN portal.[2] The levels of protein expression in normal tissue and the primary tumors of ten available cancers were plotted in terms of the Z score, which represents the standard deviations from the

median across samples. The CPTAC spectral count ratios were normalized within each sample profile and across samples. The gene expressions related to the patient pathological stages were plotted by the "Expression Analysis" module of GEPIA2 with one-way ANOVA.[3] The gene expression in stage I to IV was transformed to $\log_2$(TPM (i.e. transcripts per million)+1) for comparison. The RNA-Seq based data in tumor, normal and metastatic tissues were obtained from TCGA, GTEx and Therapeutically Applicable Research to Generate Effective Treatments (TARGET) projects (https://ocg.cancer.gov/programs/target) by using TNMplot.[4] The Kruskal-Wallis test was used to calculate the p values.

Survival prognosis analysis

The survival analyses of OGT and OGA were conducted based on the mRNA RNA-seq data across 21 tumor types from the databases including GEO, EGA, and TCGA. A web-based tool, Kaplan-Meier Plotter,[5] was used to visualize the overall survival (n=7462) and the disease-free survival (n=4420) results. The high- and low-expression groups were separated at a most significant cutoff value for separation by iterating over the variable values from the low quartile to the upper quartile and computing the Cox regression for each setting. A false discovery rate (FDR) was calculated by Benjamini-Hochberg method to correct for hypothesis testing. The significance for the comparison between the two cohorts was computed using the Cox-Mantel (log rank) test. The results with an FDR $\leqslant$ 20% and a $p < 0.05$ were reported. The hazards rate (HR) at 95% confidence interval was also calculated based on the differential descent rate of the tow cohorts.

Immune infiltration analysis

The mRNA expressions related to the tumor mutation burden (TMB) and the tumor microsatellite instability (MSI) were generated by the TCGA project and obtained through UCSCXenaShiny[6] and UCSC Xena.[7] The immune signatures, immune scores, stroma scores, and cytotoxic T cell

dysfunction were retrieved from CIBERSORT,[8] TIMER2.0,[1] and xCELL.[9] Heatmaps were based on the partial Spearman's correlation of the gene expressions across diverse cancer types.

Treatment responses analysis

Drug sensitivity of the two genes were evaluated by IC50 values compared between high and low expression groups. The data were based on the Cancer Cell Line Encyclopedia (CCLE)[10], obtained through UCSCXenaShiny[6]. The differences of drug response (IC_{50} value) for 24 anticancer drugs were compared between groups with high- and low-expression levels of OGT or OGA. We used TIMER2.0,[1] TISIDB,[11] and R package "ggplot2"[12] to visualize the Spearman correlations of the gene expression with the tumor purity and the levels of infiltration, immunoinhibitors and immunostimulators of the cancer associated fibroblasts and myeloid derived suppressor cells. We used the "biomarker evaluation" module in TIDE (tumor immune dysfunction and exclusion)[13] to predict the immunotherapy responses of OGT or OGA. The expression levels were calculated in the immune checkpoint blockade (ICB) cohorts, and the predictive power of response outcomes were compared with other published biomarkers in terms of the area under the receiver operating characteristic curve (AUC).

References

(1) Li, T.; Fu, J.; Zeng, Z.; Cohen, D.; Li, J.; Chen, Q.; Li, B.; Liu, X. S. TIMER2.0 for Analysis of Tumor-Infiltrating Immune Cells. *Nucleic Acids Res.* **2020**, *48* (W1), W509–W514. https://doi.org/10.1093/nar/gkaa407.

(2) Chandrashekar, D. S.; Karthikeyan, S. K.; Korla, P. K.; Patel, H.; Shovon, A. R.; Athar, M.; Netto, G. J.; Qin, Z. S.; Kumar, S.; Manne, U.; Creighton, C. J.; Varambally, S. UALCAN: An Update to the Integrated Cancer Data Analysis Platform. *Neoplasia N. Y. N* **2022**, *25*, 18–27. https://doi.org/10.1016/j.neo.2022.01.001.

(3) Tang, Z.; Kang, B.; Li, C.; Chen, T.; Zhang, Z. GEPIA2: An Enhanced Web Server for Large-Scale Expression Profiling and Interactive Analysis. *Nucleic Acids Res.* **2019**, *47* (W1), W556–W560. https://doi.org/10.1093/nar/gkz430.

(4) Bartha, Á.; Győrffy, B. TNMplot.Com: A Web Tool for the Comparison of Gene Expression in Normal, Tumor and Metastatic Tissues. *Int. J. Mol. Sci.* **2021**, *22* (5), 2622. https://doi.org/10.3390/ijms22052622.

(5) Nagy, Á.; Munkácsy, G.; Győrffy, B. Pancancer Survival Analysis of Cancer Hallmark Genes. *Sci. Rep.* **2021**, *11* (1), 6047. https://doi.org/10.1038/s41598-021-84787-5.
(6) Wang, S.; Xiong, Y.; Zhao, L.; Gu, K.; Li, Y.; Zhao, F.; Li, J.; Wang, M.; Wang, H.; Tao, Z.; Wu, T.; Zheng, Y.; Li, X.; Liu, X.-S. UCSCXenaShiny: An R/CRAN Package for Interactive Analysis of UCSC Xena Data. *Bioinforma. Oxf. Engl.* **2021**, *38* (2), 527–529. https://doi.org/10.1093/bioinformatics/btab561.
(7) Goldman, M. J.; Craft, B.; Hastie, M.; Repečka, K.; McDade, F.; Kamath, A.; Banerjee, A.; Luo, Y.; Rogers, D.; Brooks, A. N.; Zhu, J.; Haussler, D. Visualizing and Interpreting Cancer Genomics Data via the Xena Platform. *Nat. Biotechnol.* **2020**, *38* (6), 675. https://doi.org/10.1038/s41587-020-0546-8.
(8) Chen, B.; Khodadoust, M. S.; Liu, C. L.; Newman, A. M.; Alizadeh, A. A. Profiling Tumor Infiltrating Immune Cells with CIBERSORT. *Methods Mol. Biol. Clifton NJ* **2018**, *1711*, 243–259. https://doi.org/10.1007/978-1-4939-7493-1_12.
(9) Aran, D.; Hu, Z.; Butte, A. J. XCell: Digitally Portraying the Tissue Cellular Heterogeneity Landscape. *Genome Biol.* **2017**, *18* (1), 220. https://doi.org/10.1186/s13059-017-1349-1.
(10) Barretina, J.; Caponigro, G.; Stransky, N.; Venkatesan, K.; Margolin, A. A.; Kim, S.; Wilson, C. J.; Lehár, J.; Kryukov, G. V.; Sonkin, D.; Reddy, A.; Liu, M.; Murray, L.; Berger, M. F.; Monahan, J. E.; Morais, P.; Meltzer, J.; Korejwa, A.; Jané-Valbuena, J.; Mapa, F. A.; Thibault, J.; Bric-Furlong, E.; Raman, P.; Shipway, A.; Engels, I. H.; Cheng, J.; Yu, G. K.; Yu, J.; Aspesi, P.; de Silva, M.; Jagtap, K.; Jones, M. D.; Wang, L.; Hatton, C.; Palescandolo, E.; Gupta, S.; Mahan, S.; Sougnez, C.; Onofrio, R. C.; Liefeld, T.; MacConaill, L.; Winckler, W.; Reich, M.; Li, N.; Mesirov, J. P.; Gabriel, S. B.; Getz, G.; Ardlie, K.; Chan, V.; Myer, V. E.; Weber, B. L.; Porter, J.; Warmuth, M.; Finan, P.; Harris, J. L.; Meyerson, M.; Golub, T. R.; Morrissey, M. P.; Sellers, W. R.; Schlegel, R.; Garraway, L. A. The Cancer Cell Line Encyclopedia Enables Predictive Modeling of Anticancer Drug Sensitivity. *Nature* **2012**, *483* (7391), 603–607. https://doi.org/10.1038/nature11003.
(11) Ru, B.; Wong, C. N.; Tong, Y.; Zhong, J. Y.; Zhong, S. S. W.; Wu, W. C.; Chu, K. C.; Wong, C. Y.; Lau, C. Y.; Chen, I.; Chan, N. W.; Zhang, J. TISIDB: An Integrated Repository Portal for Tumor-Immune System Interactions. *Bioinforma. Oxf. Engl.* **2019**, *35* (20), 4200–4202. https://doi.org/10.1093/bioinformatics/btz210.
(12) Villanueva, R. A. M.; Chen, Z. J. Ggplot2: Elegant Graphics for Data Analysis (2nd Ed.). *Meas. Interdiscip. Res. Perspect.* **2019**, *17* (3), 160–167. https://doi.org/10.1080/15366367.2019.1565254.
(13) Fu, J.; Li, K.; Zhang, W.; Wan, C.; Zhang, J.; Jiang, P.; Liu, X. S. Large-Scale Public Data Reuse to Model Immunotherapy Response and Resistance. *Genome Med.* **2020**, *12* (1), 21. https://doi.org/10.1186/s13073-020-0721-z.

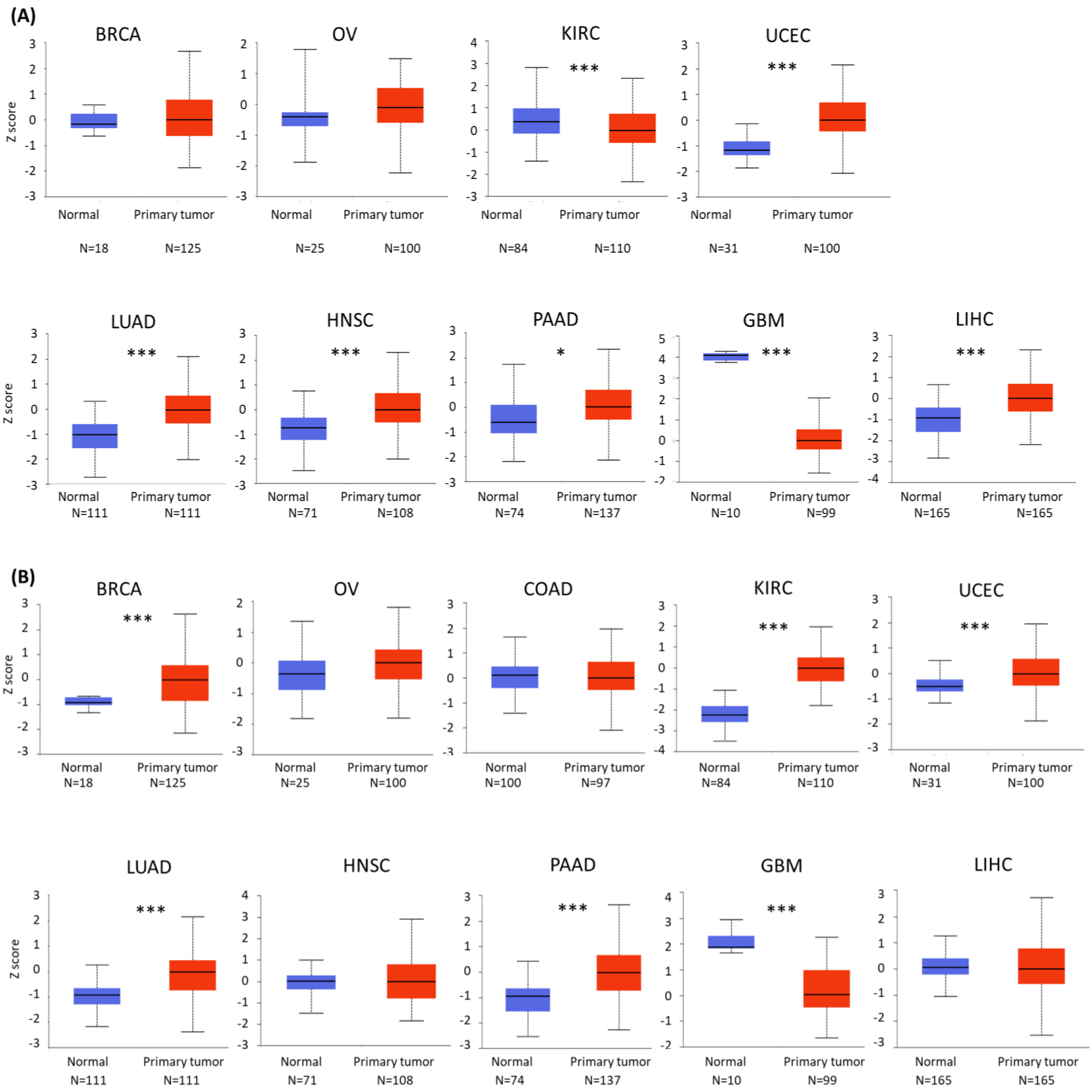

Figure S1. The protein levels of OGT (A) and MGEA5/OGA (B) in different types of cancer, with comparison performed between tumor tissue and normal tissue. $^{*}p < 0.05$, $^{**}p < 0.01$, and $^{***}p < 0.001$.

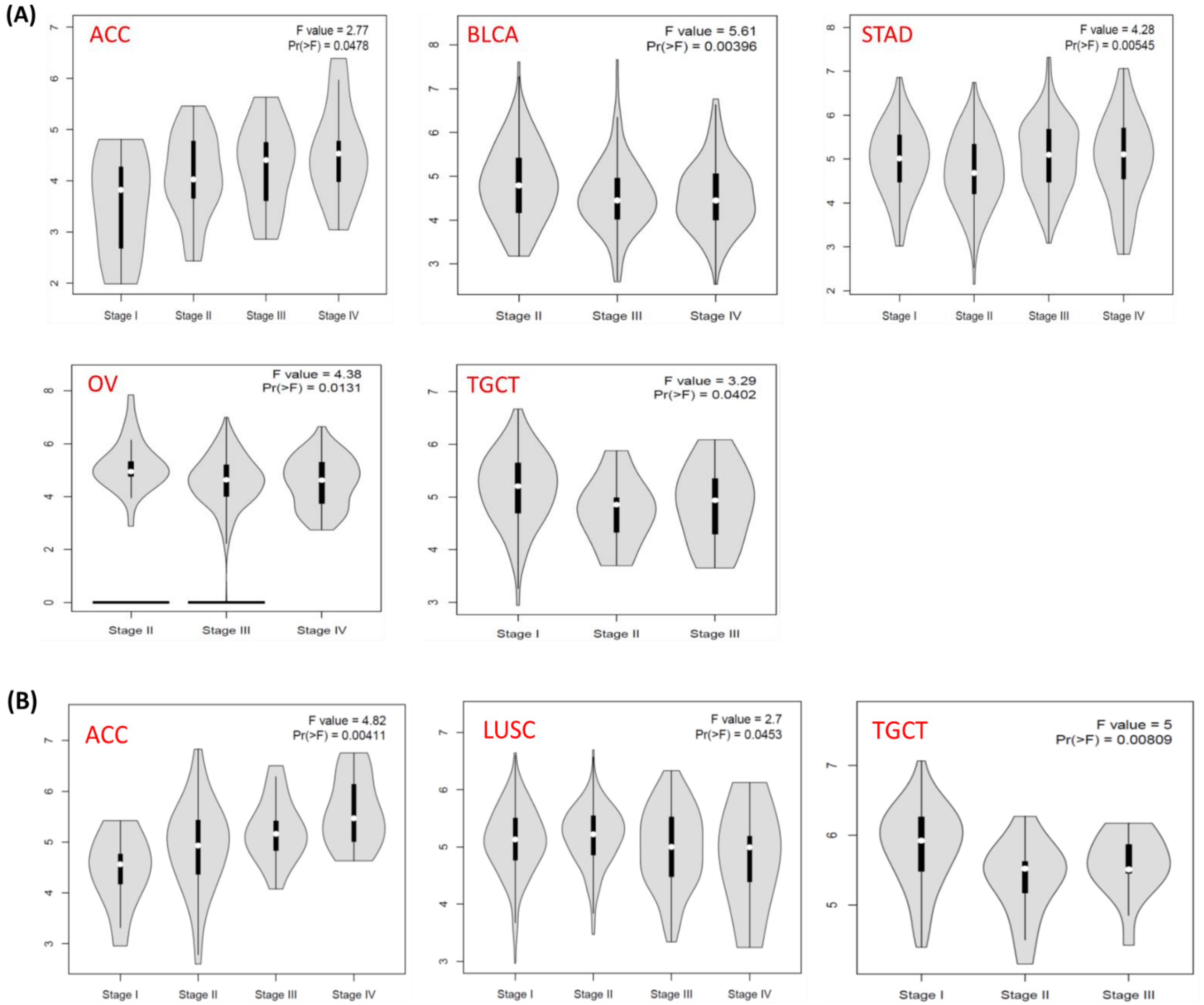

Figure S2. The expression levels of OGT (A) and MGEA5/OGA (B) in different pathological stages (stage I, stage II, stage III, and stage IV) of different types of cancer.

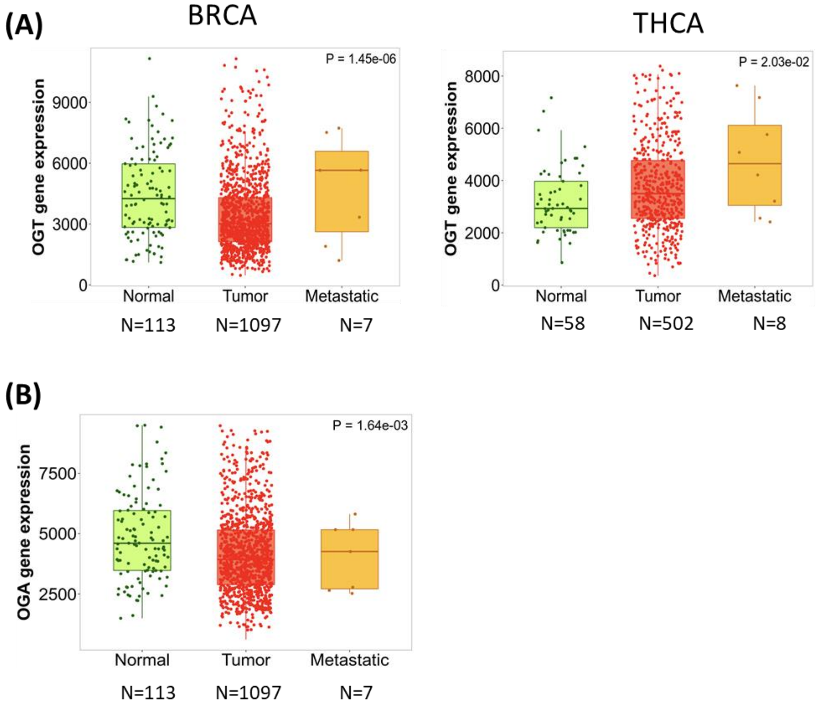

Figure S3. The expression levels of OGT (A) and MGEA5/OGA (B) between normal tissue, tumor tissue, and metastatic tissue of different types of cancer.

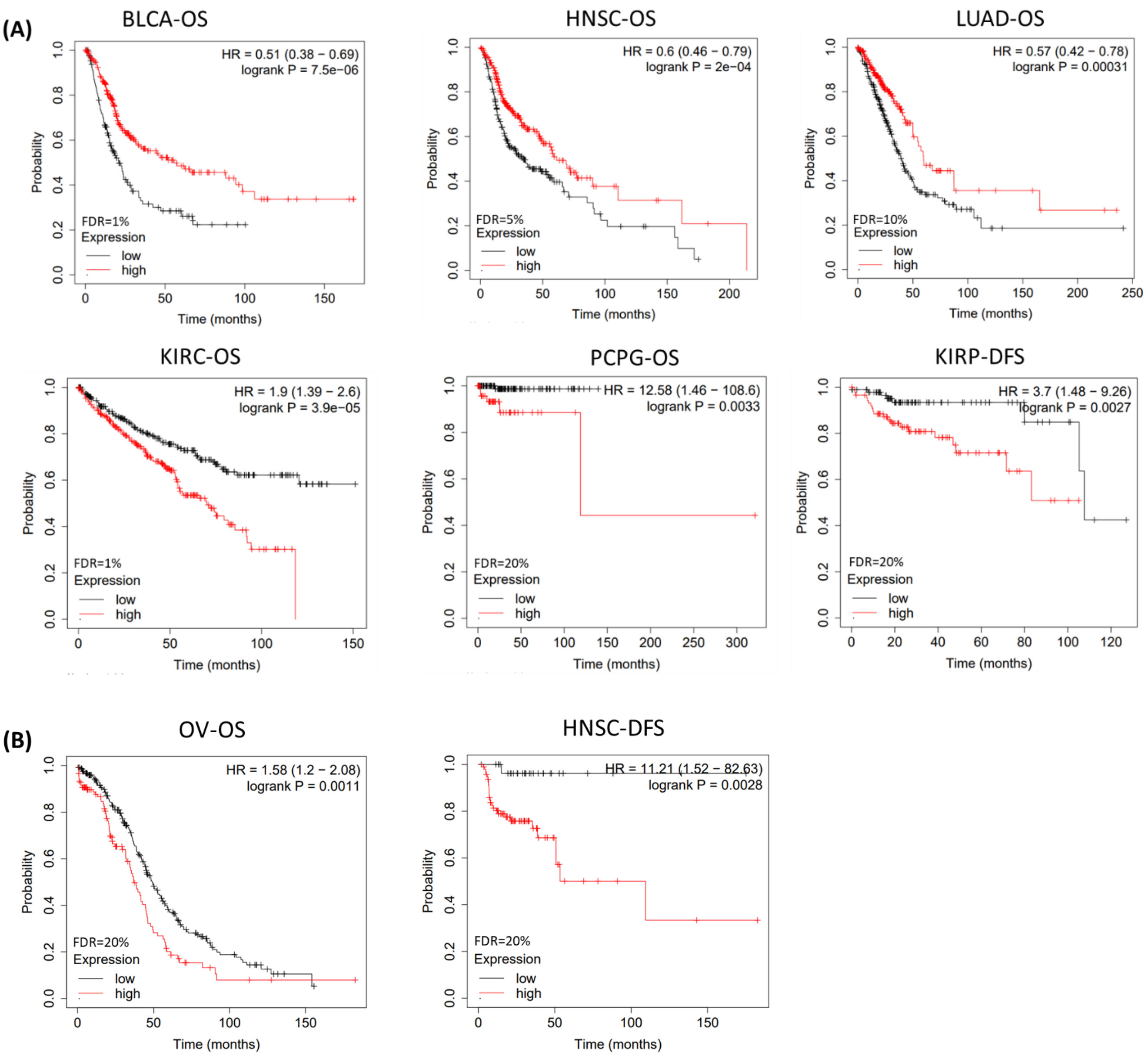

Figure S4. Correlation between OGT(A)/MGEA5(OGA, B) expression and survival of patients in different kinds of tumor. OS, overall survival; DFS, disease-free survival.

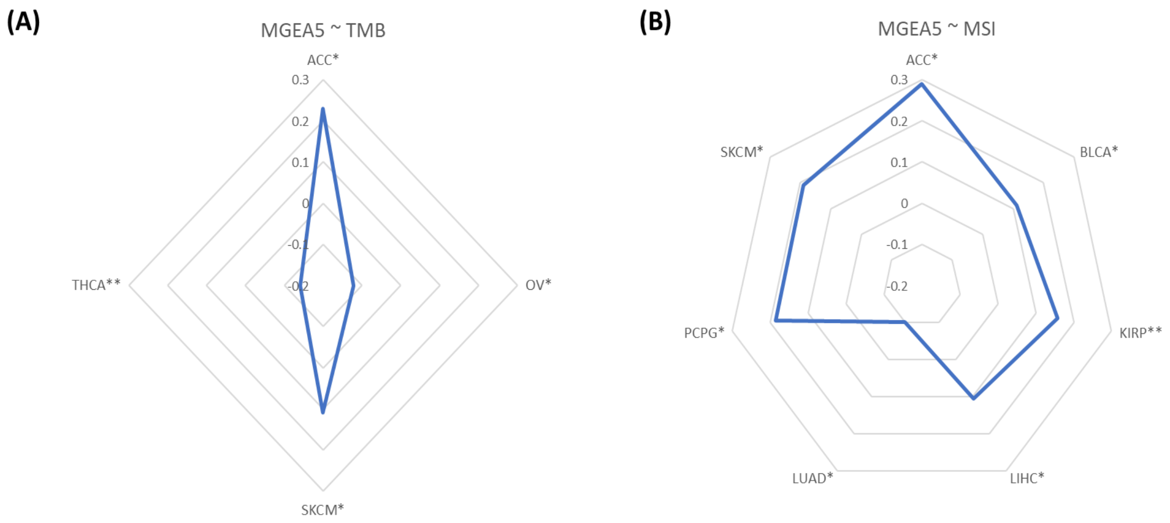

Figure S5. Correlation between of MGEA5/OGA and TMB (A) or MSI (B) in cancers, with only significant ones shown. *p < 0.05, **p < 0.01, and ***p < 0.001.

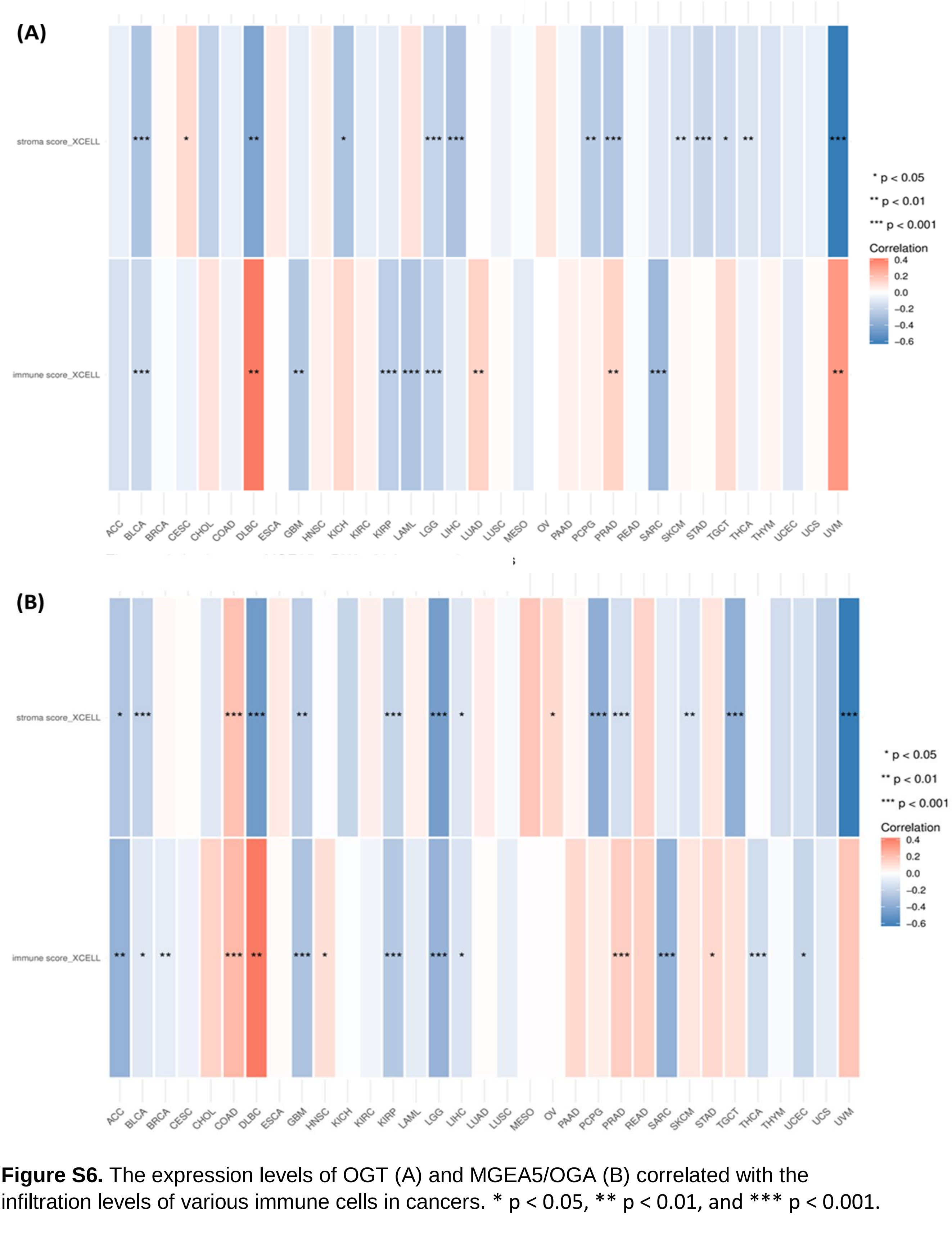

Figure S6. The expression levels of OGT (A) and MGEA5/OGA (B) correlated with the infiltration levels of various immune cells in cancers. * $p < 0.05$, ** $p < 0.01$, and *** $p < 0.001$.

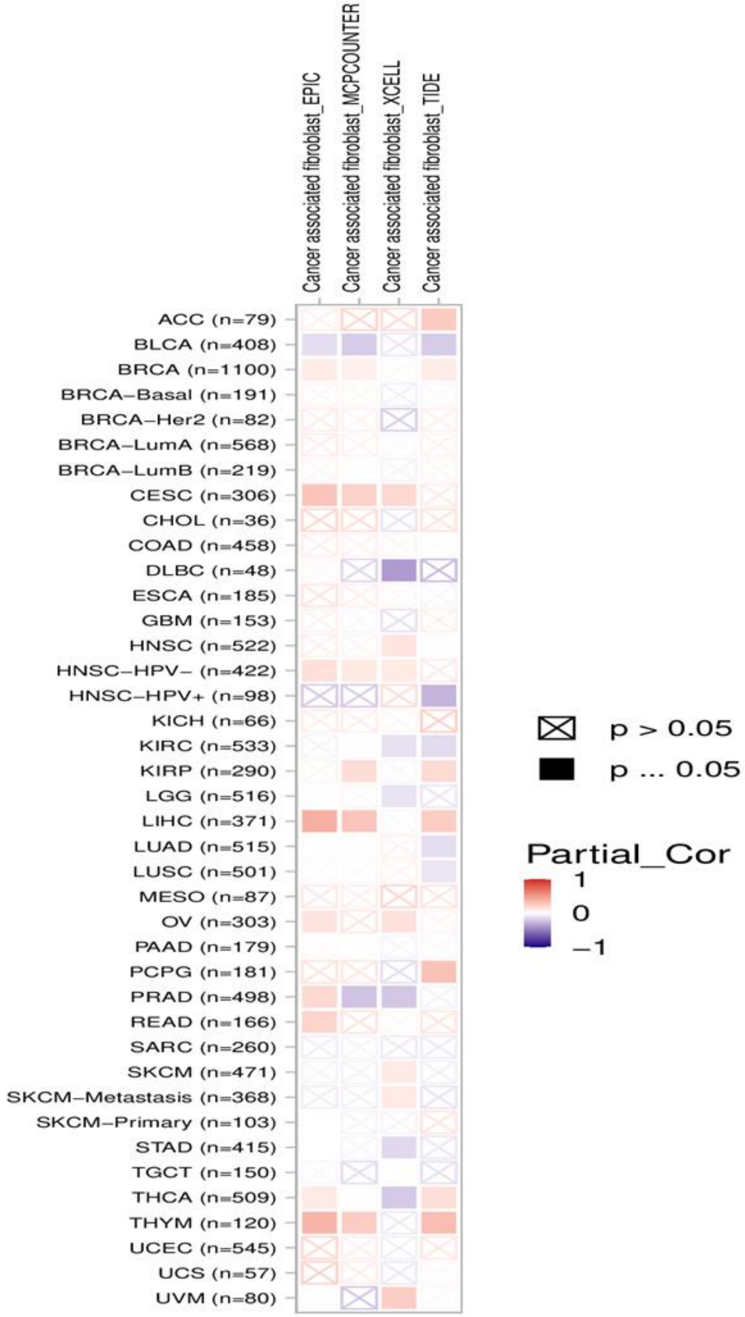

Figure S7. The expression levels of OGT with cancer-associated fibroblasts (CAFs) in cancers.

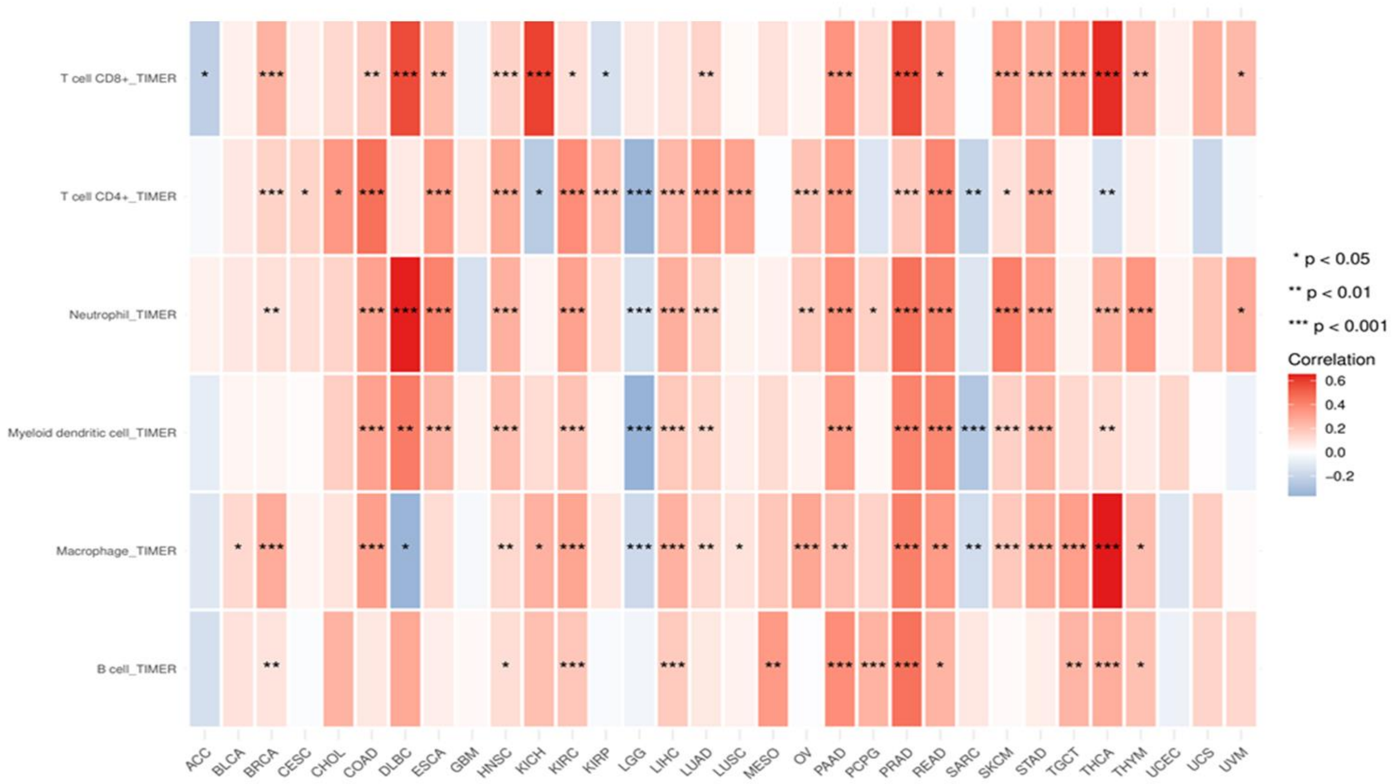

Figure S8. Expression of MGEA5/OGA with the infiltration levels of various immune cells in pan-cancer. p<0.05, ** p<0.01, *** p<0.001.

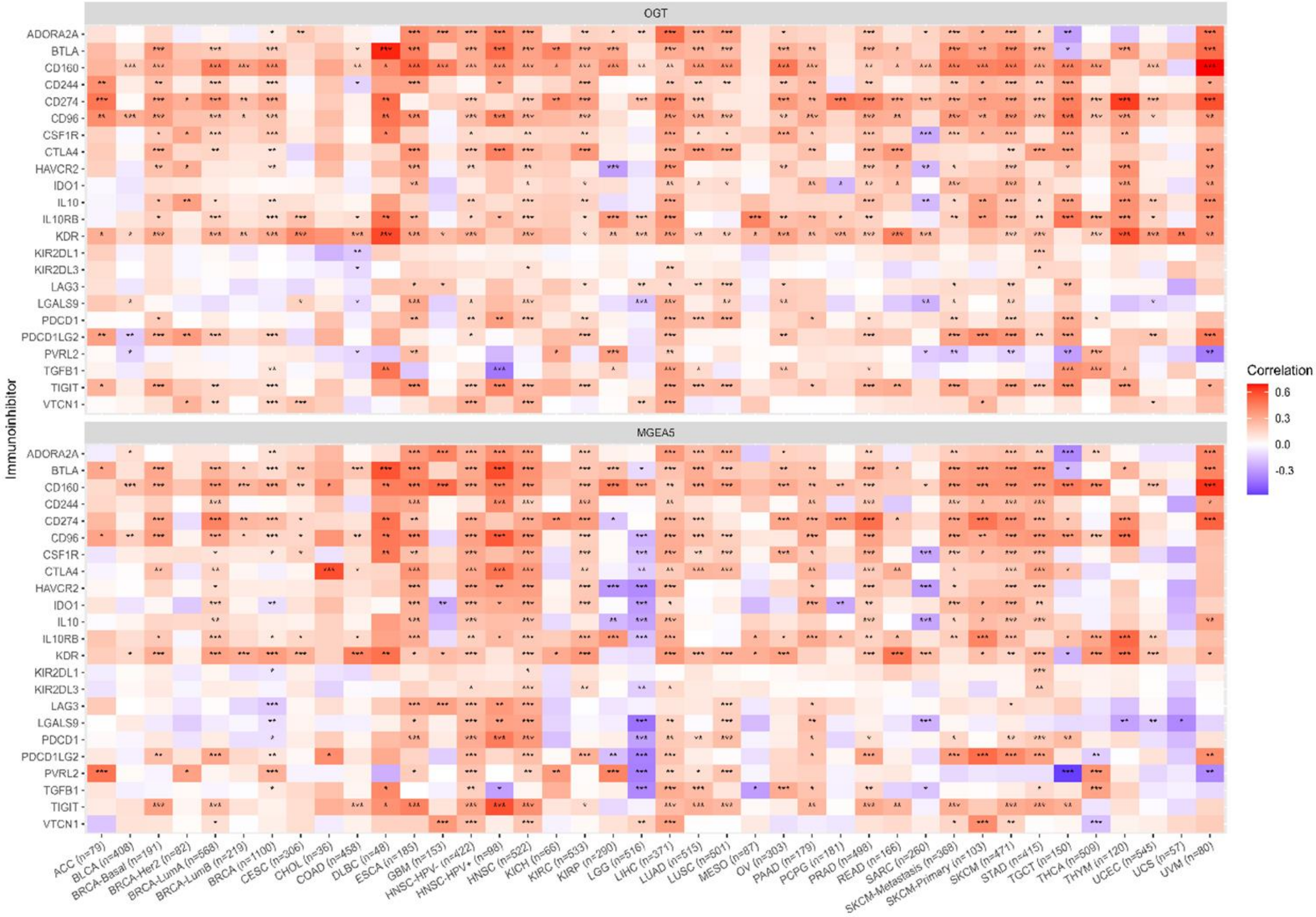

Figure S9. Correlations between the expression of OGT (top) or MGEA5/OGA (bottom) and immunoinhibitiors in pan-cancer. p<0.05, ** p<0.01, *** p<0.001.

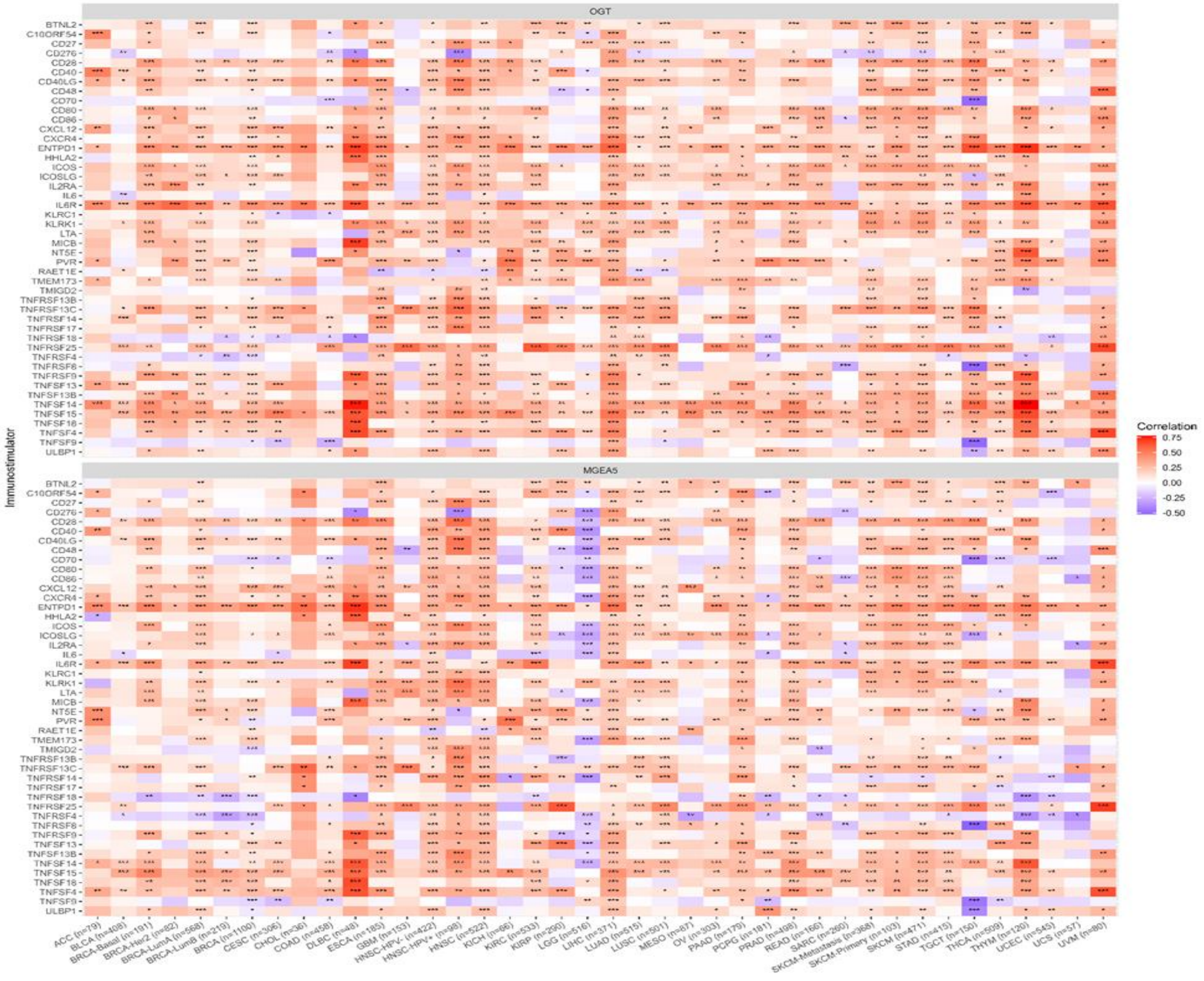

Figure S10. Correlations between the expression of OGT (top) or MGEA5/OGA (bottom) and immunostimulators in pan-cancer. p < 0.05, ** p < 0.01, *** p < 0.001.

(A)

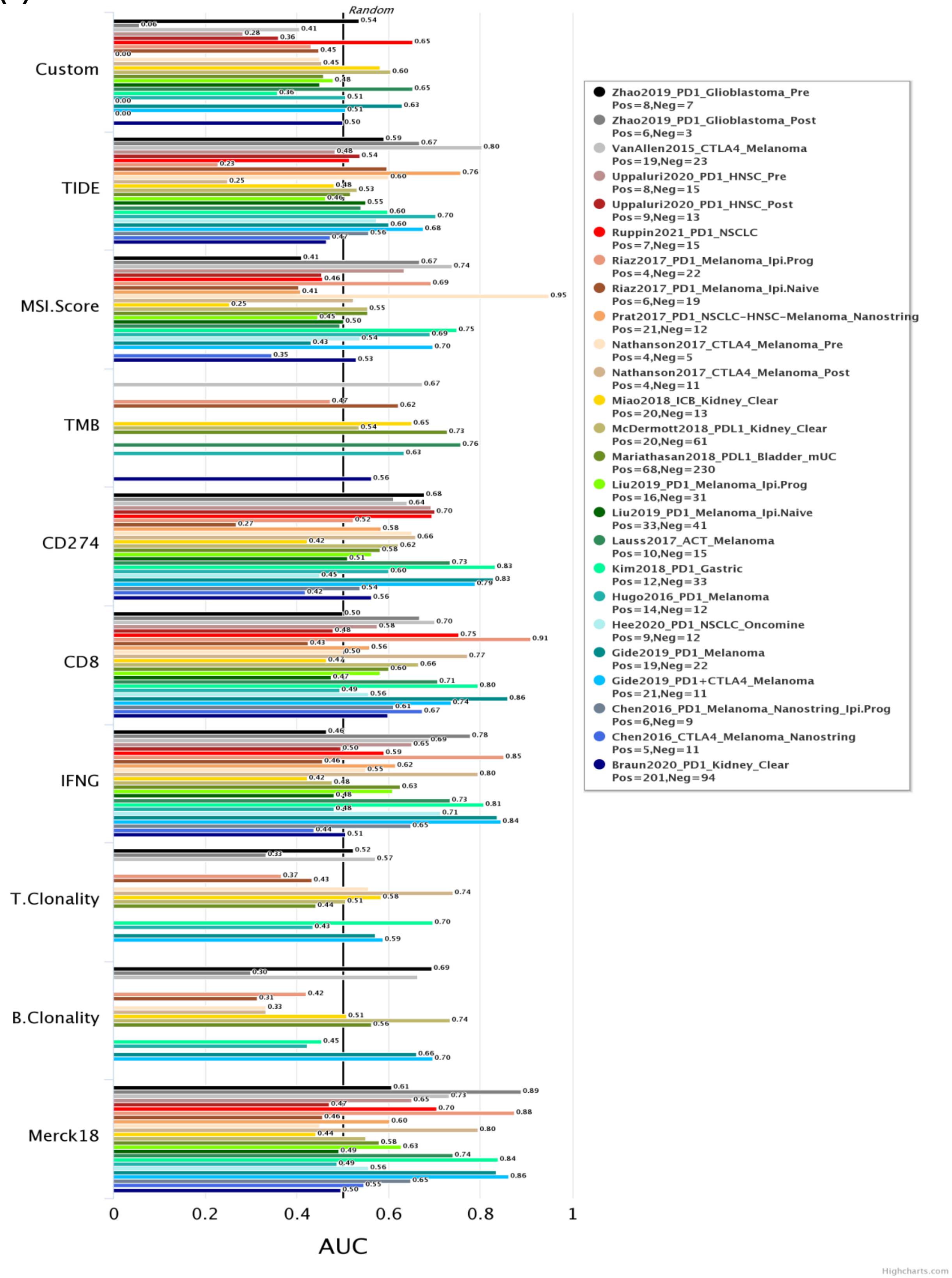
Random
Custom
TIDE
MSI.Score
TMB
CD274
CD8
IFNG
T.Clonality
B.Clonality
Merck18
0
0.2
0.4
0.6
0.8
1
AUC
Zhao2019_PD1_Glioblastoma_Pre Pos=8,Neg=7
Zhao2019_PD1_Glioblastoma_Post Pos=6,Neg=3
VanAllen2015_CTLA4_Melanoma Pos=19,Neg=23
Uppaluri2020_PD1_HNSC_Pre Pos=8,Neg=15
Uppaluri2020_PD1_HNSC_Post Pos=9,Neg=13
Ruppin2021_PD1_NSCLC Pos=7,Neg=15
Riaz2017_PD1_Melanoma_Ipi.Prog Pos=4,Neg=22
Riaz2017_PD1_Melanoma_Ipi.Naive Pos=6,Neg=19
Prat2017_PD1_NSCLC-HNSC-Melanoma_Nanostring Pos=21,Neg=12
Nathanson2017_CTLA4_Melanoma_Pre Pos=4,Neg=5
Nathanson2017_CTLA4_Melanoma_Post Pos=4,Neg=11
Miao2018_ICB_Kidney_Clear Pos=20,Neg=13
McDermott2018_PDL1_Kidney_Clear Pos=20,Neg=61
Mariathasan2018_PDL1_Bladder_mUC Pos=68,Neg=230
Liu2019_PD1_Melanoma_Ipi.Prog Pos=16,Neg=31
Liu2019_PD1_Melanoma_Ipi.Naive Pos=33,Neg=41
Lauss2017_ACT_Melanoma Pos=10,Neg=15
Kim2018_PD1_Gastric Pos=12,Neg=33
Hugo2016_PD1_Melanoma Pos=14,Neg=12
Hee2020_PD1_NSCLC_Oncomine Pos=9,Neg=12
Gide2019_PD1_Melanoma Pos=19,Neg=22
Gide2019_PD1+CTLA4_Melanoma Pos=21,Neg=11
Chen2016_PD1_Melanoma_Nanostring_Ipi.Prog Pos=6,Neg=9
Chen2016_CTLA4_Melanoma_Nanostring Pos=5,Neg=11
Braun2020_PD1_Kidney_Clear Pos=201,Neg=94
Highcharts.com

(B)

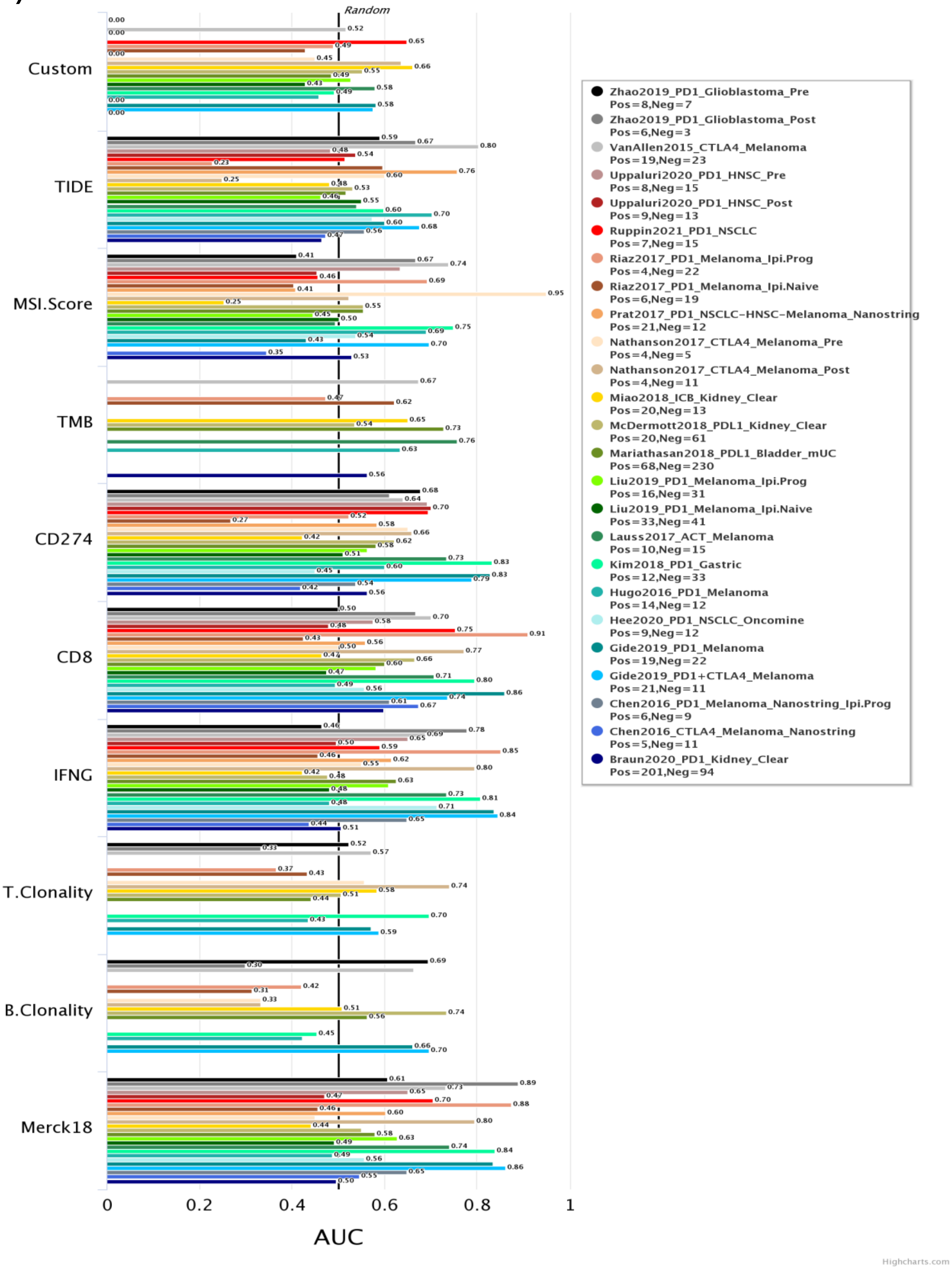

Figure S11. Correlations between the expression levels of OGT (A) or MGEA5/OGA (B) and immunotherapy responses.

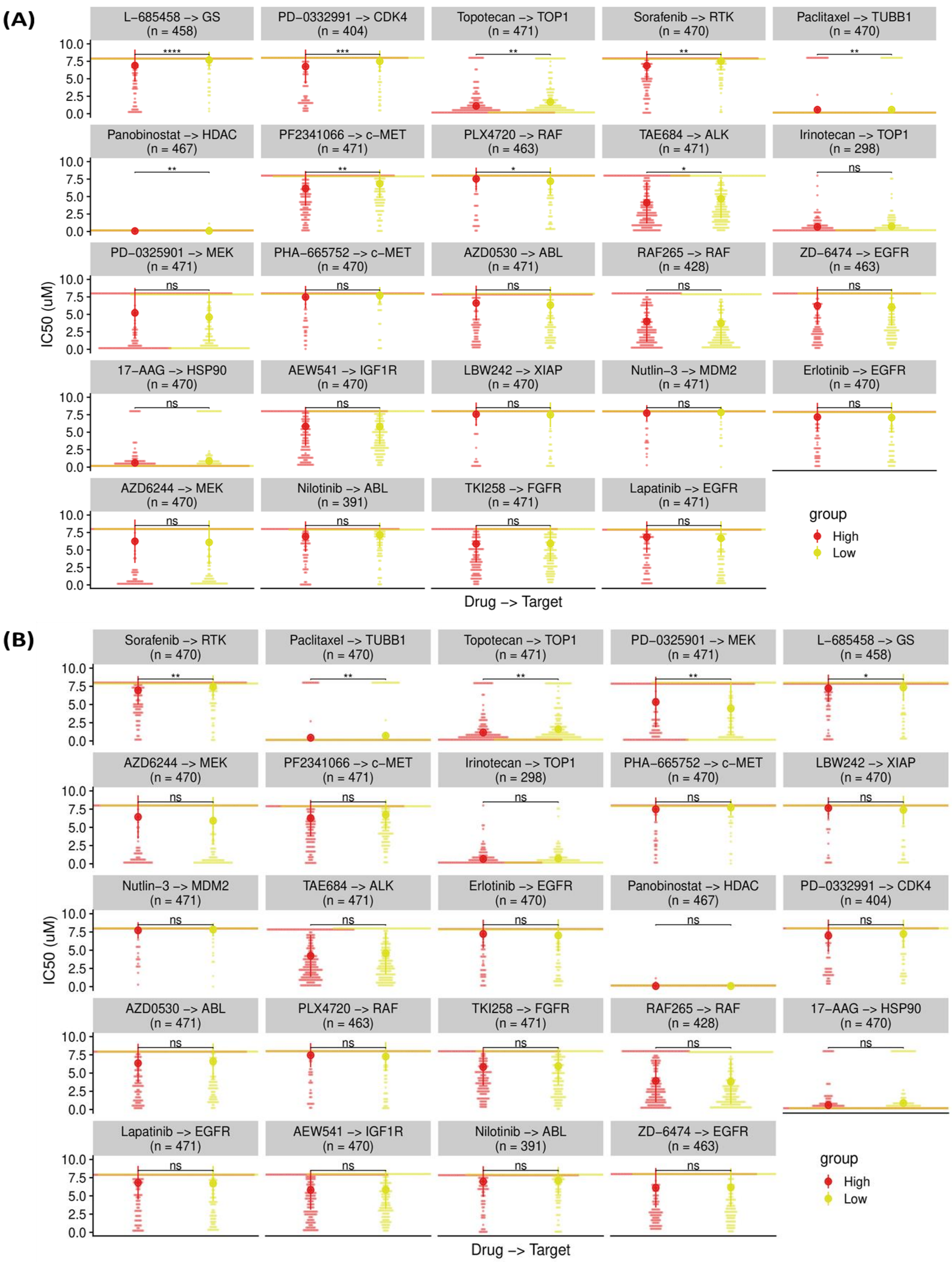

Figure S12. Correlations between the expression levels of OGT (A) or MGEA5/OGA (B) and drug responses in cancers. * p < 0.05, ** p < 0.01, and *** p < 0.001.
